# Dietary Fiber Intake (Supplemental or Dietary Pattern Rich in Fiber) and Diabetic Kidney Disease: A Systematic Review of Clinical Trials

**DOI:** 10.3390/nu11020347

**Published:** 2019-02-06

**Authors:** Cláudia Mesquita de Carvalho, Luiza Azevedo Gross, Mirela Jobim de Azevedo, Luciana Verçoza Viana

**Affiliations:** Endocrine Division, Hospital de Clínicas de Porto Alegre, Universidade Federal do Rio Grande do Sul. Rua Ramiro Barcelos 2350, Prédio 12, 4º andar, Porto Alegre-RS 90035-003, Brazil; luizaazevedogross@gmail.com (L.A.G.); vercoza@yahoo.com (L.V.V.)

**Keywords:** vegetarian diet, diabetic nephropathy, albuminuria, glomerular filtration rate, systematic review

## Abstract

Fiber intake is associated with better glycemic control being an important non-pharmacological treatment for diabetes (DM). We hypothesize that a dietary fiber intake can bring benefits to diabetic kidney disease (DKD), improving renal outcomes. This systematic review aimed to evaluate the effect of dietary fiber (supplemental or dietary pattern rich in fiber) on DKD. We searched six databases to identify clinical trials that reported fiber intake and renal outcomes (albuminuria, proteinuria, estimated glomerular filtration rate (eGFR) dialysis) in patients with DM. From 1814 studies, 48 papers were fully evaluated. In the end, seven trials (161 patients, aged 58.3 years, 49% females) were included. The studies were organized into three categories (vegetarian, Dietary Approaches to Stop Hypertension (DASH) diet, and fiber supplement), two evaluated supplements and five dietary patterns. Vegetarian diet reduced albuminuria in three trials, two in patients with type 1 DM and one in patients with type 2 DM; and one study demonstrated a change in the eGFR in type 1 DM. The individual quality of the studies was low/uncertain. A vegetarian dietary pattern may have a beneficial effect on these renal outcomes. However, the individual effect of the intake of fiber on DKD not was possible to be evaluated.

## 1. Introduction

Diabetes Mellitus (DM) is a growing worldwide epidemic. Approximately, 425 million adults are affected by this chronic disease [1]. Most of the financial burden of DM is related to management of its complications, and chronic kidney disease (CKD) is the most expensive and debilitating [2]. Cardiovascular disease (CVD) is a frequent cause of mortality in patients with type 2 DM [3], and it is well established that CKD is a risk factor for CVD [4]. Among patients with type 2 DM and coronary artery disease, mortality rates were progressively higher in patients with mild and moderate CKD compared with patients with preserved estimated glomerular filtration rate (eGFR) [5].

Nutritional gaps in the knowledge of dietary management of DKD include amount and quality of carbohydrates, protein, and fat intake [6]. It is known that fiber intake is associated with better glycemic control [7] and cardiovascular risk reduction [8]. Table 1 shows an overview of dietary fiber recommendations. The American Diabetes Association (ADA) [9] recommends that patients with DM should consume at least 14 grams of fiber for each 1000 kcals daily and it suggests that carbohydrate intake from vegetables, fruits, legumes, and whole grains, with an emphasis on foods higher in fiber and lower in glycemic load, is preferred over other sources of sugar [10]. The American Heart Association (AHA) also endorses healthy dietary patterns rich in fiber to prevent CVD, such as Dietary Approaches to Stop Hypertension (DASH) and Mediterranean diets [11]. In its latest edition, Dietary Guidelines for Americans [12] reinforced the idea of fiber consumption through specific foods and patterns.

There is no precise recommendation about fiber intake or dietary patterns rich in fiber for patients with CKD [6,13,14]. Since the effect of dietary fiber on renal outcomes is not well established, and dietary fiber appears to be an important non-pharmacological treatment for DM and CVD, it is necessary to better understand and further investigate its effects on diabetic kidney disease (DKD). We hypothesize that a dietary fiber intake can bring benefits to DKD, through improve renal outcomes. Therefore, this systematic review aimed to evaluate the effect of dietary fiber (supplemental or dietary pattern rich in fiber) on DKD.

## 2. Materials and Methods

This systematic review was carried out using a protocol constructed according to the Cochrane Handbook recommendations [15], and it was reported in accordance with the Preferred Reporting Items for Systematic Reviews and Meta-Analyses (PRISMA) statement [16] (PROSPERO CRD42017072535).

### 2.1. Data Sources and Searches

We searched databases from Medline, Embase, ClinicalTrials.gov register, Scopus, Web of Science, and Cochrane databases to identify interventional clinical trials that reported dietary fiber intake (supplemental or dietary pattern rich in fiber) and renal outcomes (albuminuria, proteinuria, eGFR, and dialysis) in patients with DM, up to January 2018. 

The initial search comprised the terms diabetes, dietary fiber, diabetic nephropathy, albuminuria, proteinuria, diabetic kidney disease, eGFR, renal replacement therapy, kidney failure chronic, and related entry terms. The complete Medline search strategy is presented in Appendix A, available in the Supporting Material for this article online. All potentially eligible studies were reviewed, regardless of the primary outcome or language.

### 2.2. Study Selection

Only interventional clinical trials conducted on patients with diabetes (type 1 or type 2 DM) with at least one high fiber group were included in the present review. Dietary fiber intake (supplemental or dietary pattern rich in fiber) was compared with conventional or low-fiber diets. Dietary intervention must have lasted at least four weeks. We excluded studies with non-clinical trials design (cohort, cross-sectional, case-control, and review studies), with the same dietary intervention in all studied groups, without dietary information, or without data about renal outcomes. 

### 2.3. Data Extraction and Quality Assessment

All citations retrieved from electronic databases were imported into the EndNote Program. Two reviewers (CMC, LAG) independently analyzed the titles and abstracts of every paper retrieved from the literature search to identify potentially eligible studies. The full text of the remaining papers was obtained for further examination. Disagreements were solved by a third reviewer (LVV). 

Data of included studies were independently extracted by the same two reviewers using a standardized form. Extracted data included: first author’s name, year of publication, sample size, study design, trial duration, general characteristics of participants (the type of DM, age, gender, body mass index, HbA1c, hypertension or blood pressure), intervention characteristics and outcomes of interest. A detailed description of the type of diet (actual intake or prescribed diet), total energy, macronutrients, and fiber content was documented for interventions and comparators. 

In this review, we used renal outcomes definitions provided by authors of the included studies. In general, DKD is diagnosed based on the persistence of albuminuria and/or reduced eGFR in the absence of signs or symptoms of other primary causes of kidney damage [17]. End-of-study and baseline means or statistical dispersion for outcomes were extracted. 

Methodological quality of studies was measured according to the Cochrane Collaboration’s Handbook [15]. Biases were classified into six domains: selection, performance, detection, attrition, reporting, and other [15,18]. The "other" chosen domain was the assessment of dietary compliance. The risk of bias was independently analyzed by two reviewers (CMC, LAG) for each domain, and was classified as high, low, or unclear. Regarding dietary compliance, the risk was classified as "low" if the study described the method of a dietary adherence. The performance domain (blinding of participants and personnel) was not possible to evaluate in studies that have dietary intervention (dietary pattern).

## 3. Results

### 3.1. Literature Search

**Figure 1:**
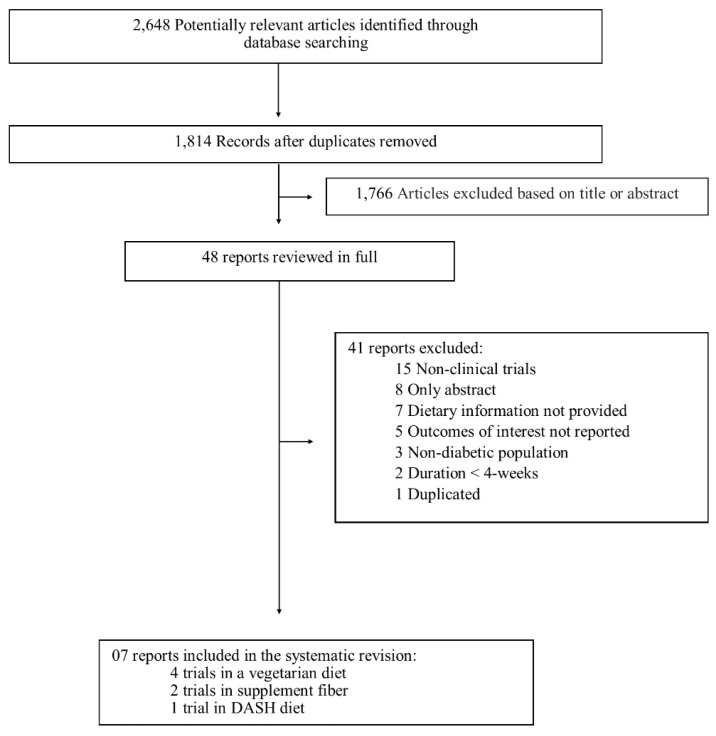
Flow diagram of the literature search to identify clinical trials evaluating the effect of dietary fiber on renal outcomes (albuminuria, eGFR) of patients with diabetes.

We identified 1814 studies in database searches. Of them, 1766 were excluded based on title and abstract, leaving 48 articles for further full-text evaluation. We excluded 41 studies (Appendix B), mainly due to lack of dietary or renal outcomes information, non-clinical trials design or only abstract available. As a result, seven interventional clinical trials were included in the current systematic review (Figure 1).

### 3.2. Study Characteristics

The seven interventional clinical trials comprised 161 patients with DM, age from 20 to 74 years (mean 58.3 years) and 49% females. HbA1c was reported in six studies (range 6.9–8.7%). Good glycemic control, defined as HbA1c below 7% was achieved in two trials. Three trials had renal outcomes as a primary outcome [19,20,21], and only one study included patients with established renal disease (macroalbuminuria) [19]. All trials included few patients (range 8–49), had a small duration of the intervention (four to twelve weeks), and most studies were conducted in Brazil [19,22,23]. Four trials had a parallel design [22,23,24,25], and three showed a crossover design [19,20,21]. Two studies included patients with type 1 DM [20,21]. Dialysis and proteinuria were not reported in any included studies. Two studies [23,24] did not describe eGFR, one study did not report albuminuria [25], and one study reported fractional albumin clearance [21]. Table 2 shows the complete description of the included trials.

Didactically, the studies were organized into three sections by type of dietary intervention: “vegetarian diet” (*n* = 4) [19,20,21,24], “fiber supplement” (*n* = 2) [22,25], and “DASH diet” (*n* = 1) [23]. Most interventions were compared to usual diet. Mean fiber intake in the intervention was 24 g/day (range: 20–27 g/day) and 16 g/day (range: 14–20 g/day) in the control group. Given the wide heterogeneity among studies regarding the type and form of dietary interventions, as well as the assessed outcomes, and the small number of studies identified in the literature, we could not perform a meta-analysis of the extracted data. The following sections provide additional detail on the included studies, grouped by type of intervention.

### 3.3. Vegetarian diet

#### 3.3.1. Type 1 DM

Two interventional clinical trials, including 17 adult patients (age 32 to 46 years), 59% females, assessed the vegetarian diets compared to a usual diet in patients with type 1 DM [20,21]. The studies were conducted in Greece [20] and the United Kingdom [21]. The intervention period ranged from four to eight weeks, and both had crossover clinical trials. Total fiber intake was 0.2 to 0.4 g/kg/day in the intervention versus 0.1 to 0.3 g/kg/day in the control group. 

In the study conducted by Kontessis et al. [20], diets were isocaloric and with the same quantity of protein, but the intervention group contained exclusively vegetable protein and a mean fiber intake of 0.2 g/kg/day versus 0.1 g/kg/day in the control group. eGRF and albuminuria were significantly lower for the intervention group.

In the study of Jibani et al. [21], the intervention consisted of a predominantly vegetarian diet, with animal protein fraction limited to approximately 30% of the total protein intake. Median actual fiber consumption was 0.4 g/kg/day in the intervention versus 0.3 g/kg/day in the control. There was no change in eGFR, but the fractional albumin clearance was significantly lower in the vegetarian group compared with the conventional diet. 

#### 3.3.2. Type 2 DM

We found two randomized clinical trials that assessed the effect of a vegetarian diet on patients with type 2 DM [19,24]. The studies were conducted in the United States [24] and Brazil [19], the sample size ranged from 11 to 17 patients, with a mean age of 57 years, 36% were females, and the study duration ranged from four to twelve weeks. In the interventions, patients ingested 26.5 g of total fiber versus 20g in the control groups (conventional diets).

In the pilot trial of Nicholson et al. [24], the intervention consisted of a low-fat vegan diet compared to a conventional diet, and there was no difference in the albuminuria between groups. The study performed by Mello et al. [19] was a crossover trial that evaluated the effects of a lactovegetarian compared with a chicken based or usual diet. No difference was observed in the eGFR between the vegetarian and usual or chicken diet. However, lactovegetarian and chicken-based diets reduced albuminuria by the same amount compared with usual diet. The quantity of fiber was greater in the lactovegetarian group, but the protein intake was smaller in the lactovegetarian group compared with chicken and usual diets. 

### 3.4. Fiber Supplement

Two randomized clinical trials conducted in patients with type 2 DM used 10g of a soluble fiber supplement (guar gum or inulin) [22,25]. The studies were conducted in Brazil [22] and Iran [25] and the sample size ranged from 40 to 49 patients with a mean age of 54.5 years, 71% females, and mostly obese patients (BMI: 30.3 kg/m^2^). The intervention period ranged from six weeks to two months. 

In the study of Dall’Alba et al. [22], total fiber intake was 24 g/day (10 g/day of guar gum supplement) compared with 16 g/day in the control taken for six weeks. There was no difference in the eGFR and albuminuria between groups. 

The study of Farhangi et al. [25] included only women, and the intervention group received a 10 g/day chicory inulin as supplemental fiber, and the control group received 10 g/day maltodextrin for two months. No information was available about total fiber intake. No changes in eGFR values had been observed. 

### 3.5. DASH Diet

Only one study evaluated the benefit of DASH diet and physical activity for four weeks compared with a diet based on ADA recommendations in patients with type 2 DM [23]. The study was conducted in Brazil and included 40 patients with DM and hypertension, mean age 62 years old, 55% females. The total fiber consumed in the intervention group was 20 versus 14 g/day in the control group. There were no differences in albuminuria between groups at the end-of-the study. 

### 3.6. Methodological Quality Assessment of Studies

None of the included studies satisfied all areas established by the Cochrane Handbook [15]. In general, the quality of the studies was low or uncertain. The selection bias domain was not possible to be evaluated in one study, because it was a non-randomized clinical trial [21]. Three studies provided a detailed description about random sequence generation [22,23,25], while only one study described the method used for allocation concealment [25]. The blinding of participants was described in two studies (dietary fiber supplement) [22,25]. The intervention providers were blinded to the group assignment in only one study [25]. One study had a loss of follow-up greater than 20% in the run-in period (~57%). On reporting bias, one study presented the outcome eGFR without having previously described it in the registry [19]. All studies properly presented the item “diet or supplement adherence". Table 3 shows the complete assessment of the methodological quality of the included studies.

## 4. Discussion

Diet is the cornerstone treatment for patients with DM and as far as we know this is the first systematic review that attempts to shows benefits of a consumption of dietary fiber on renal outcomes in patients with DM. Among the seven included studies, only the vegetarian dietary pattern was associated with beneficial kidney outcomes: three studies showed a reduction of albuminuria (two conducted in patients with type 1 DM and one in patients with type 2 DM) and one study demonstrated a change in the eGFR in patients with type 1 DM. 

As we already know, the pathogenesis of diabetic nephropathy has complex mechanisms including the effect of high glucose, endothelial dysfunction inflammation, renin-angiotensin system activation, reactive oxygen species, increase of advanced glycation end-product, and glomerular hyperfiltration [26]. Dietary fiber plays an important role in glycemic control [7,27] and regardless of the source (food or supplements) fiber exhibits hypoglycemic actions in patients with type 2 DM [28,29]. Some studies have shown that good glycemic control reduces albuminuria [30,31,32] in patients with DM and dietary fiber intake was found to be associated with a reduced risk of albuminuria in a cross-sectional study [33]. Recently, a Japanese randomized clinical trial showed that a diet higher in fiber was able to improve endothelial function, possibly by a reduction of glucose excursions, in patients with type 2 DM [34]. 

In the general population, consumption of fiber-rich foods can reduce serum creatinine levels [35,36] and may increase eGFR in CKD patients without DM [36]. A prospective study showed that high fiber intake, mainly from legumes and vegetables, was related to lower incidence of CKD after six years of follow-up. For every additional 5 g/day of fiber intake, there was an 11% reduction in risk of CKD [37]. In fact, the precise effects of dietary fiber consumption on renal function are not well known, but healthy dietary patterns with high fiber are associated with lower mortality in people with kidney disease [38].

In our systematic review, a dietary pattern rich in fiber: Vegetarian diet was the only category associated with a reduction in albuminuria in both type 1 and type 2 DM patients and reduction in eGFR in a group of patients with type 1 DM and possible hyperfiltration. It is worth noting that protein intake was lower in two out of three of these studies. It has been suggested that plant-based proteins may exert beneficial effects on blood pressure, protein loss in urine, and GFR, and reduce renal tissue damage preventing the progression of CKD when compared to animal proteins [39]. It is difficult to isolate the effect of a single nutrient, in this case, dietary fiber from protein. A vegetarian dietary pattern is usually richer in fibers, but it is lower in animal protein, which may be more suitable for these patients and exert beneficial glomerular effects [40]. Higher eGFR has been demonstrated in patients with a normal renal function on an animal protein diet in comparison with a person on a vegetable-based diet [41,42]. 

Bioavailability of dietary proteins in plant-based diets is diverse. The content of phosphorus, amino acids types, and advanced glycation end products (AGEs) is distinguished in animal and plant diets. Plant-based sources of protein tend to have the less bioavailable form of phosphorus as phytate compared to organic phosphorus found in processed foods as well as in animal sources of protein. The mechanisms by which plant-based diets may reduce DKD progression may be related to the form of phosphorus, AGEs, a reduction in blood pressure by decreasing sodium, an increase in fiber leading to improved glycemic control, or from bioactive compounds in soy protein-based diets, such as isoflavones [43]. Additionally, dietary protein also delivers an increased acid load per nephron providing a non-hemodynamic mechanism promoting renal injury through the induction of endothelin and aldosterone in response to increased nephron ammonia generation. In the presence of proteinuria, increased dietary protein alters glomerular permselectivity, increasing urinary albumin loss [44].

In our review, no other dietary pattern had favorable effects on renal outcomes. DASH diet was not able to reduce eGFR or albuminuria in patients with type 2 DM similar to the results of Jacobs et al. in patients without DM [45]. However, in two large, prospective, long-term studies, adherence to DASH diet was associated with protection against eGFR decline [46,47]. No clinical trial specifically designed to evaluate the effects of a Mediterranean diet in patients with DM was identified in our database search. In a recent cohort study, a Mediterranean dietary pattern was associated with a decreased risk of CKD in patients with and without DM [48]. A Mediterranean diet was too evaluated in a randomized clinical trial, PREDIMED (*PREvencion con DIeta MEDiterranea*) study. However, two subgroup analyses that evaluated only patients with type 2 DM, showed no difference in nephropathy between Mediterranean groups compared to a low-fat diet [49,50]. These studies were not included in this systematic review due to them not presenting the dietary fiber intake.

Limitations of our systematic review are the small number of studies, with a small sample size, few ethnic groups represented among the participants, and short follow-up time (no more than twelve weeks). This may limit the effect of any dietary intervention in renal outcomes, particularly because CKD is a slowly progressive disease. Many years may be necessary for the development of kidney damage. In fact, DKD was present in only one study in our systematic review [19]. Regarding the quality of the included studies, lack of description of trial characteristics made the quality analysis unclear in several domains. Fiber dietary intake in included studies was lower than recommended by most dietary guidelines (38 g/day for men and 25 g/day women) [9,51,52]. In addition, we could not establish an independent fiber effect on renal outcomes since most included studies evaluated eating patterns. On the other hand, dietary patterns seem to be more important than a single nutrient and offer a more practical application in public health promotion since it is easier for people to adopt dietary patterns instead of specific nutrients from their diets [53]. 

Evidence of benefits of dietary fiber on renal outcomes in patients with DM is still limited, and more precise indications of the amount, duration, and the type of fiber intake to achieve these goals are needed. Regrettably, we could not evaluate the individual effect of different fiber sources (legumes, vegetables, or fruits) on the outcomes. We know that all fibers are not the same and sources of fibers carry other nutrients (i.e., vitamins and minerals) that on their own already have positive effects on health. Available data extrapolated from the general population, show that fiber intake is likely protective against CKD progression and mortality [27,37,54], and every effort should be made to encourage higher fiber intake in the CKD population. Unfortunately, we could not reach a definitive conclusion regarding the beneficial effect of fiber in DKD in our systematic review. Still, larger, longer, better design trials are needed to evaluate the effect of fiber on DKD. 

A vegetarian dietary pattern may have a beneficial effect on these renal outcomes. However, the individual effect of the intake of fiber on DKD was not possible to be evaluated on our systematic review. New randomized trials are needed to reach a definitive conclusion.

## Figures and Tables

**Table 1:** Characteristics of dietary patterns and fiber recommendations.

Dietary Patterns/ Guidelines Recommendations	Main Foods	Nutrients Characteristics
DASH diet ^1,2,4^	Includes vegetables, fruits, whole grains, fat-free or low-fat dairy products, fish, poultry, beans, nuts, and vegetable oils; <25% dietary intake from fat; low in sweets, sugar-sweetened beverages, and tropical oils.	Low in saturated fats and cholesterol Rich in fiber Rich in protein
Vegetarian diet ^2,3, 4^	Includes whole grains, vegetables, fruits, legumes, nuts, seeds, soy and, if desired, dairy products, and eggs. Does not include meat, fowl or seafood, or products containing those foods.	Rich in fiber Rich in *n*-6 fatty acids Rich in vegetable protein
Mediterranean diet ^1,2,4^	Includes fruits, vegetables, whole grains, beans, nuts, seeds, seafood, olive oil; low to moderate amounts of poultry, and dairy products, with little red meat; low to moderate wine consumption (optional).	Rich in fiber Rich in monounsaturated and polyunsaturated fat
Guidelines recommendations	^1^ AHA: Rich in fiber ^2^ American Guideline: 14 g/1000kcal ^5^ European Guideline: 25 g/day ^4^ ADA: 14g/1000 kcal or 25 g/day women, 38 g/day men ^6^ KDOQI/^7^KDIGO: no specific recommendation	

ADA = American Diabetes Association; AHA = American Heart Association; KDOQI = Kidney Disease Outcomes Quality Initiative; KDIGO = Kidney Disease Outcomes; Quality Initiative. ^1^ American Heart Association—Guideline on Lifestyle Management to Reduce Cardiovascular Risk, 2013; ^2^ Dietary Guidelines for Americans, 2015; ^3^ Position of the American Dietetic Association: Vegetarian Diets, 2015; ^4^ American Diabetes Association, 2014/2018; ^5^ Scientific Opinion on Dietary Reference Values for carbohydrates and dietary fiber, 2010; ^6^ KDOQI—Chronic Kidney Disease Evidence-Based Nutrition Practice Guideline, 2010; ^7^ Diabetic Kidney Disease—A clinical update from Kidney Disease: KDIGO.

**Table 2:** Characteristics of the studies evaluating the effect of fiber intake on renal outcomes (albuminuria and eGFR) in patients with diabetes.

Author Year Country	Sample Characteristics	Study Design	Diet Characteristics	Renal Outcomes
Type 1 diabetes				
Vegetarian diet				
Jibani 1991 *n* = 8 United Kingdom	Females: 37.5% Hypertension: 25% Age: 46 (22–70) years HbA1c: NA BMI: NA Withdrawals: 20% Duration: 8-weeks	Crossover clinical trial Washout: 8-weeks	Intervention (*n* = 10): vegetarian diet * Energy: 32 (23–34) kcal/kg/day CHO: 3.4 (2.3–4.2) g/kg/day; Prot: 1.0 g/kg/day; Lip: 1.2 g/kg/day Total fiber: 0.4 g/kg/day Soluble fiber: NA Insoluble fiber: NA Control (*n* = 10): conventional diet * Energy: 29 (15–35) kcal/kg /day CHO: 3.7 (2.6–4.6) g/kg/day; Prot: 1.4 g/kg/day; Lip: 1.4 g/kg/day Total fiber: 0.3 g/kg/day Soluble fiber: NA Insoluble fiber: NA	Intervention eGFR (mL/min/1.73 m^2^) § Final: 109 (48–163) Fractional albumin clearance (x10^−4^) ** Final: 87 (23–829) Control eGFR (mL/min/1.73 m^2^) Final: 109 (45–134) Fractional albumin clearance (x10^−4^) Final: 188 (58–810)
Kontessis 1995 *n* = 9 Greece	Females: 77.8% Hypertension: 0% Age: 32 (20–48) years HbA1c: 6.7 (5.1–8.4)% BMI: 23.8 (20.6–27.8) kg/m^2^ Withdrawals: 0% Duration: 4-weeks	Randomized crossover clinical trial Washout: ≥1-week	Intervention (*n* = 9): vegetal protein diet * Energy: 22.8 ± 3.8 kcal/kg/day CHO: 2.8 ± 0.7 g/kg/day; Prot: 0.95 ± 0.3 g/kg/day; Lip: 0.9 ± 0.1 g/kg/day Total fiber: 0.2 ± 0.03 g/kg/day Soluble fiber: NA Insoluble fiber: NA Control (*n* = 9): animal protein diet * Energy: 23.3 ± 3.7 kcal/kg /day CHO: 2.4 ± 0.65 g/kg/day; Prot: 1.1 ± 0.3 g/kg/day; Lip: 0.95 ± 0.1 g/kg/day Total fiber: 0.1 ± 0.1 g/kg/day Soluble fiber: NA Insoluble fiber: NA	Intervention eGFR (mL/min/1.73 m^2^) ** Basal: 110 (88–129) Final: 89.9 ± 4.1 Albuminuria (mg/24 h) ** Final: 10.4 (1.3–22.5) Control eGFR (mL/min/1.73 m^2^) Basal: 110 (88–129) Final: 105.6 ± 5.1 Albuminuria (mg/24 h) Final: 17.1 (4.1–44.5)
Type 2 diabetes				
Vegetarian diet				
Nicholson 1999 *n* = 11 USA	Females: 45.5% Hypertension: 81.8% Age: 54.3 (34–74) years HbA1c: 8.2 ± 1.5% BMI: NA Withdrawals: 15.4% Duration: 12-weeks	Randomized clinical trial	Intervention (*n* = 7): low fat vegan diet * Energy: 1409 ± 549 kcal/day CHO: 75 ± 4.4%; Prot: 14 ± 1.6%; Lip: 11 ± 4.7% Total fiber: 26 ± 8.2 g/day Soluble fiber: NA Insoluble fiber: NA Control (*n* = 4): conventional diet * Energy: 1526 ± 314 kcal/day CHO: 51 ± 3.5%; Prot: 18 ± 1.4%; Lip: 31 ± 2.4% Total fiber: 20 ± 2.7 g/day Soluble fiber: NA Insoluble fiber: NA	Intervention Albuminuria (mg/24 h) § Basal: 434.8 ± 565.5 Final: 155.2 ± 182.6 Control Albuminuria (mg/24 h) Basal: 82.9 ± 114.6 Final: 169.2 ± 298
Mello 2006 *n* = 17 Brazil	Females: 17.6% Hypertension: 47% Age: 59 ± 11 years HbA1c: 7.6 ± 2.6% BMI: 26.2 ± 2.6 kg/m^2^ Withdrawals: 57.5% Duration: 4-weeks	Randomized crossover clinical trial Washout: 4-weeks	Intervention (*n* = 17): lactovegetarian diet * Energy: 1634 ± 451 kcal/day CHO: 58.7 ± 6.8%; Prot: 11.6 ± 1.5%; Lip: 29.5 ± 6.8% Total fiber: 27 ± 8.1 g/day Soluble fiber: NA Insoluble fiber: NA Control (*n* = 17): usual diet * Energy: 1901 ± 480 kcal/day CHO: 46.9 ± 6.7%; Prot: 21.9 ± 3.4%; Lip: 30.8 ± 6.3% Total fiber: 20±7.5 g/day Soluble fiber: NA Insoluble fiber: NA	Intervention eGFR (mL/min/1.73 m^2^) § Final: 81.9 ± 25.3 Albuminuria (mg/24 h) ** Final: 332.5 (111.1–1449) Control eGFR (mL/min/1.73 m^2^) Final: 81.8 ± 22.2 Albuminuria (mg/24 h) Final: 453.6 (324.4–1774.4)
Fiber supplement				
Dall’Alba 2013 *n* = 44 Brazil	Females: 38.6% Hypertension: 93.2% Age: 62 ± 9.7 years HbA1c: 6.9 ± 0.8% BMI: 29.8 ± 3.7 kg/m^2^ Withdrawals: 4.3% Duration: 6-weeks	Randomized clinical trial	Intervention (*n* = 23): 10 g guar gum supplement * Energy: 1700 ± 439 kcal/day CHO: 184.2 ± 28.1 g/day; Prot: 81.5 ± 15.4 g/day; Lip: 61.5 ± 10.2 g/day Total fiber: 24.3 ± 5.4 g/day Soluble fiber: 14.8 ± 1.9 g/day Insoluble fiber: 9.5 ± 3.6 g/day Control (*n* = 21): control group * Energy: 1553 ± 371 kcal/day CHO: 191.9 ± 27.3 g/day; Prot: 86.3 ± 12 g/day; Lip: 58.3 ± 12.8 g/day Total fiber: 15.7 ± 6.3 g/day Soluble fiber: 5.2 ± 1.9 g/day Insoluble fiber: 10.5 ± 4.7 g/day	Intervention eGFR (mL/min/1.73 m^2^) § Basal: 84.8 ± 16.6 Final: 85 ± 16.2 Albuminuria (mg/24 h) ‡ Basal: 6.8 (3–17.5) Final: 6.2 (3–9.5) Control eGFR (mL/min/1.73 m^2^) § Basal: 89.2 ± 16.7 Final: 89 ± 17.4 Albuminuria (mg/24 h) § Basal: 6.7 (3–19.3) Final: 7.6 (3–15.8)
Farhangi 2016 *n* = 49 Iran	Females: 100% Hypertension: NA Age: 48.3 ± 8.8 years HbA1c: 8.3 ± 0.9% BMI: 30.8 ± 3.9 kg/m^2^ Withdrawals: 9.3% Duration: 2-months	Randomized clinical trial	Intervention (*n* = 27): 10g chicory inulin supplement Energy: NA CHO: NA; Prot: NA; Lip: NA Total fiber: NA Soluble fiber: 10 g/day Insoluble fiber: NA Control (*n* = 22): placebo Energy: NA CHO: NA; Prot: NA; Lip: NA Total fiber: NA Soluble fiber: NA Insoluble fiber: NA	Intervention eGFR (mL/min/1.73 m^2^) § Basal: 86.3 ± 14 Final: 84.3 ± 13.6 Control eGFR (mL/min/1.73 m^2^) Basal: 85.3 ± 13.5 Final: 82.1 ± 16.1
DASH diet				
Paula 2015 *n* = 40 Brazil	Females: 55% Hypertension: 100% Age: 62.2 ± 8.4 years HbA1c: 8.7 ± 1.8% BMI: 29.4 ± 3.4 kg/m^2^ Withdrawals: 0% Duration: 4-weeks	Randomized clinical trial	Intervention (*n* = 20): DASH diet * Energy: 1585 ± 321 kcal/day CHO: 47.1 ± 7.3%; Prot: 23.5 ± 6.7%; Lip: 29.4 ± 5.8% Total fiber: 20.1 ± 4.3 g/day Soluble fiber: 6.1 ± 2.1 g/day Insoluble fiber: 12.9 ± 2.9 g/day Control (*n* = 20): ADA recommendations * Energy: 1752 ± 299 kcal/day CHO: 39.3 ± 9.9%; Prot: 23 ± 3.8%; Lip: 36.8 ± 8% Total fiber: 14.1 ± 4.8 g/day Soluble fiber: 4.7 ± 2.1 g/day Insoluble fiber: 11 ± 5.3 g/day	Intervention Albuminuria (mg/24 h) § Basal: 41.6 (22.1–185.8) Final: 31.8 (10.2–132.7) ‡ Control Albuminuria (mg/24 h) Basal: 43.5 (18.5–194.4) Final: 33.4 (11.2–119.6)

Abbreviators: ADA = American Diabetic Association; BMI = body mass index; kcal = kilocalories; CHO = carbohydrates; DASH = Dietary Approaches to Stop Hypertension; Egfr = estimated glomerular filtration rate; Lip = Lipids; NA = Not available; NS = Not significant; Prot = Protein. * actual intake ** *p* < 0.05 for the effect of diet between groups; ‡ *p* < 0.05 for the effect of diet within group; § Not significant.

**Table 3:** Assessment of methodological quality or risk of bias item for each included study.

	Selection Bias		Performance Bias	Detection Bias	Attrition Bias	Reporting Bias	Other Bias
	Random sequence generation	Allocation Concealment	Blinding of participant and personnel	Blinding of outcome assessment	Incomplete outcome data	Selective reporting	Diet/supplement adherence
Jibani, 1991	NA *	NA *	NA *	Uncertain	Low	Uncertain	Low
Kontessis, 1995	Uncertain	Uncertain	NA *	Uncertain	Uncertain	Uncertain	Low
Nicholson, 1999	Uncertain	Uncertain	NA *	Uncertain	Low	Uncertain	Low
Mello, 2006	Uncertain	Uncertain	NA *	Uncertain	High	Uncertain	Low
Dall’Alba, 2013	Low	Uncertain	High	Uncertain	Low	Low	Low
Paula, 2015	Low	Uncertain	NA *	Uncertain	Low	Low	Low
Farhangi, 2016	Low	Low	Low	Low	Low	High	Low

Abbreviators: * NA = not applicable for this type of study. Adapted from Cochrane Collaboration’s tool.4.

## Appendix A. MEDLINE Search Strategy for the Systematic Review

((((“Diabetes Mellitus” [Mesh] OR (Diabetes Mellitus) OR “Diet, Diabetic” [Mesh] OR (Diet, Diabetic) OR (Diabetic Diets) OR (Diets, Diabetic) OR (Diabetic Diet) OR "Diabetes Mellitus, Type 2” [Mesh] OR (Diabetes Mellitus, Type 2) OR (Noninsulin-Dependent Diabetes Mellitus) OR (Type 2 Diabetes Mellitus) OR (Diabetes Mellitus, Type II) OR (Stable Diabetes Mellitus) OR (Diabetes Mellitus, Stable) OR (Slow-Onset Diabetes Mellitus) OR (Diabetes Mellitus, Slow Onset) OR (Diabetes Mellitus, Slow-Onset) OR (Diabetes Mellitus, Noninsulin Dependent) OR (Non-Insulin-Dependent Diabetes Mellitus) OR (Diabetes Mellitus, Non-Insulin-Dependent) OR (Diabetes Mellitus, Non Insulin Dependent) OR (Maturity-Onset Diabetes) OR (Diabetes Mellitus, Adult-Onset) OR (Diabetes Mellitus, Noninsulin-Dependent) OR (Adult-Onset Diabetes Mellitus) OR (Diabetes Mellitus, Adult Onset) OR (NIDDM) OR (Diabetes Mellitus, Maturity Onset) OR (Diabetes Mellitus, Maturity-Onset) OR (Diabetes Mellitus, Ketosis-Resistant) OR (Diabetes Mellitus, Ketosis Resistant) OR (Ketosis-Resistant Diabetes Mellitus) OR (Type II DM) OR (Type 2 DM) OR "Diabetes Mellitus, Type 1" [Mesh] OR (Diabetes Mellitus, Type 1) OR (Diabetes Mellitus, Brittle) OR (Brittle Diabetes Mellitus) OR (Diabetes Mellitus, Insulin-Dependent) OR (Diabetes Mellitus, Insulin Dependent) OR (Insulin-Dependent Diabetes Mellitus) OR (Diabetes Mellitus, Juvenile-Onset) OR (Diabetes Mellitus, Juvenile Onset) OR (Juvenile-Onset Diabetes Mellitus) OR (Diabetes Mellitus, Ketosis-Prone) OR (Diabetes Mellitus, Ketosis Prone) OR (Ketosis-Prone Diabetes Mellitus) OR (Juvenile-Onset Diabetes) OR (Diabetes, Juvenile-Onset) OR (Juvenile Onset Diabetes) OR (Diabetes Mellitus, Type I) OR (Diabetes Mellitus, Sudden-Onset) OR (Diabetes Mellitus, Sudden Onset) OR (Diabetes Mellitus, Sudden Onset) OR (Sudden-Onset Diabetes Mellitus) OR (Type 1 Diabetes Mellitus) OR (Diabetes Mellitus, Insulin-Dependent, 1) OR (Insulin-Dependent Diabetes Mellitus 1) OR (Insulin Dependent Diabetes Mellitus 1) OR (Type 1 Diabetes) OR (Diabetes, Type 1) OR (IDDM) OR (Diabetes, Autoimmune) OR (Autoimmune Diabetes)))) AND ("Dietary Fiber" [Mesh] OR (Dietary Fiber) OR (Fiber, Dietary) OR (Dietary Fibers) OR (Fibers, Dietary) OR (Roughage) OR (Roughages) OR (Wheat Bran) OR (Bran, Wheat) OR (Brans, Wheat) OR (Wheat Brans) OR "Whole Grains" [Mesh] OR (Whole Grains) OR (Grain, Whole) OR (Grains, Whole) OR (Whole Grain) OR (Whole Grain Cereals) OR (Cereal, Whole Grain) OR (Cereals, Whole Grain) OR (Grain Cereal, Whole) OR (Grain Cereals, Whole) OR (Whole Grain Cereal) OR (Cereal fiber) OR “Plants, Edible” [Mesh] OR (Plants, Edible) OR (Edible Plant) OR (Edible Plants) OR (Plant, Edible) OR (Food Plants) OR (Food Plant) OR (Plant, Food) OR (Plants, Food) OR “Vegetables” [Mesh] OR (Vegetables) OR (Vegetable) OR “Fruit” [Mesh] OR (Fruit) OR (Fruits) OR (Plant Aril) OR (Arils, Plant) OR (Aril, Plant) OR (Plant Arils) OR (Fiber) OR (Soluble fiber) OR (Total fiber) OR (Insoluble fiber) OR “Diet, Vegetarian”[Mesh] OR (Diet, Vegetarian) OR (Diets, Vegetarian) OR (Vegetarian Diets) OR (Vegetarian Diet) OR (Vegetarianism) OR “Diet, Vegan” [Mesh] OR (Diet, Vegan) OR (Diets, Vegan) OR (Vegan Diets) OR (Vegan Diet) OR (Veganism) OR (Diet, Dash) OR (Diets, Dash) OR (Dash Diet) OR (Dash Diets) OR (Dash) OR (Dietary Approaches to Stop Hypertension) OR “Diet, Mediterranean”[Mesh] OR (Diet, Mediterranean) OR (Mediterranean Diet) OR (Diets, Mediterranean) OR “Inulin”[Mesh] OR (Inulin) OR “Psyllium”[Mesh] OR (Psyllium) OR (Gum, Ispaghule) OR (Ispaghula) OR (Guar gum) OR (Gum guar)))) AND ((“Diabetic Nephropathies” [Mesh] OR (Diabetic Nephropathies) OR (Nephropathies, Diabetic) OR (Nephropathy, Diabetic) OR (Diabetic Nephropathy) OR (Diabetic Kidney Disease) OR (Diabetic Kidney Diseases) OR (Kidney Disease, Diabetic) OR (Kidney Diseases, Diabetic) OR (Diabetic Glomerulosclerosis) OR (Kimmelstiel-Wilson Syndrome) OR (Kimmelstiel Wilson Syndrome) OR (Syndrome, Kimmelstiel-Wilson) OR (Kimmelstiel-Wilson Disease) OR (Kimmelstiel Wilson Disease) OR (Nodular Glomerulosclerosis) OR (Glomerulosclerosis, Nodular) OR (Glomerulosclerosis, Diabetic) OR “Albuminuria”[Mesh] OR (Albuminuria) OR (Albuminurias) OR “Proteinuria”[Mesh] OR (Proteinuria) OR (Proteinurias) OR (Microalbuminuria) OR (Macroalbuminuria) OR (Diabetic Renal Disease) OR (Increased urinary albumin excretion) OR “Glomerular Filtration Rate”[Mesh] OR (Glomerular Filtration Rate) OR (Filtration Rate, Glomerular) OR (Filtration Rates, Glomerular) OR (Glomerular Filtration Rates) OR (Rate, Glomerular Filtration) OR (Rates, Glomerular Filtration) OR (Chronic Kidney Disease) OR (Renal Replacement Therapy[Mesh]) OR (Renal Replacement Therapy) OR “Dialysis”[Mesh] OR (Dialysis) OR (Dialyses) OR “Renal Dialysis”[Mesh] OR (Renal Dialysis) OR (Hemodialysis) OR (Hemodialyses) OR “Kidney Failure, Chronic”[Mesh] OR (Kidney Failure, Chronic) OR (End-Stage Kidney Disease) OR (Disease, End-Stage Kidney) OR (End Stage Kidney Disease) OR (Kidney Disease, End-Stage) OR (Chronic Kidney Failure) OR (End-Stage Renal Disease) OR (Disease, End-Stage Renal) OR (End Stage Renal Disease) OR (Renal Disease, End-Stage) OR (Renal Disease, End Stage) OR (Renal Failure, End-Stage) OR (End-Stage Renal Failure) OR (Renal Failure, End Stage) OR (Renal Failure, Chronic) OR (Chronic Renal Failure) OR (ESRD) OR (ESKD) OR “Renal Insufficiency, Chronic”[Mesh] OR (Renal Insufficiency, Chronic) OR (Chronic Renal Insufficiencies) OR (Chronic Renal Insufficiencies) OR (Renal Insufficiencies, Chronic) OR (Chronic Renal Insufficiency) OR (Kidney Insufficiency, Chronic) OR (Chronic Kidney Insufficiency) OR (Chronic Kidney Insufficiencies) OR (Kidney Insufficiencies, Chronic) OR (Chronic Kidney Diseases) OR (Chronic Kidney Disease) OR (Disease, Chronic Kidney) OR (Diseases, Chronic Kidney) OR (Kidney Disease, Chronic) OR (Kidney Diseases, Chronic) OR (Chronic Renal Diseases) OR (Chronic Renal Disease) OR (Disease, Chronic Renal) OR (Diseases, Chronic Renal) OR (Renal Disease, Chronic) OR (Renal Diseases, Chronic))).

## Appendix B. Excluded Studies of the Systematic Review (*n* = 41)

**Author**	**Year**	**Periodic**	**Reasons to exclusion**
Paisey	1984	Diabetes Care	Non-clinical trials
Parillo	1984	Minerva Endocrinologica	Duration <4-weeks
Barsotti	1987	Infusionstherapie und Klinische Ernährung	Only abstract
Parillo	1988	American Journal of Clinical Nutrition	Duration <4-weeks
Barsotti	1988	Contributions of Nephrology	Only abstract
Naumova	1990	Vŭtreshni Bolesti	Only abstract
Barsotti	1991	American Journal of Nephrology	Non-diabetic population
Metcalf	1993	Clinical Chemistry	Outcomes of interest not reported
Hadfield	1993	Practical Diabetes	Non-clinical trials
Oldrizzi	1994	Journal of the American Society of Neprhology	Dietary information not provided
Citro	1998	Minerva Endocrinologica	Outcomes of interest not reported
GSEDNu	2006	Journal of Diabetes and Complications	Non-clinical trials
Brunori	2007	American Journal of Kidney Diseases	Non-diabetic population
Cámara	2008	Revista Espanhola de Salud Publica	Outcomes of interest not reported
Glover	2009	Food Hydrocolloids	Outcomes of interest not reported
Teixeira	2010	Diabetes	Outcomes of interest not reported
Gong	2011	Diabetologia	Dietary information not provided
Lin	2011	American Journal of Kidney Diseases	Dietary information not provided
Sun	2011	Journal of Chinese Clinical Medicine	Only abstract
Reddy	2012	Kidney Research Clinical Practice	Non-clinical trials
Dunkler	2013	JAMA Internal Medicine	Non-clinical trials
Chang	2013	American Journal of Kidney Diseases	Non-clinical trials
Tirosh	2013	Diabetes Care	Only abstract
Dans	2013	Phillipine Journal of Internal med	Only abstract
Khatri	2014	Clinical Journal of the American Society of Nephrology	Non-clinical trials
Hsu	2014	Clinical Nutrition	Non-clinical trials
Xu	2014	Clinical Journal of the American Society of Nephrology	Non-clinical trials
Díaz-López	2015	Diabetes Care	Dietary information not provided
Villarini	2015	Annali di Igiene	Non-diabetic population
Lee	2015	Nephrology Dialysis Transplantation	Non-clinical trials
Nazha	2015	Nephrology Dialysis Transplantation	Only abstract
Rebholz	2016	American Journal of Kidney Diseases	Duplicated
Dunkler	2016	American Journal of Kidney Diseases	Non-clinical trials
Piccoli	2016	BMC Nephrology	Dietary information not provided
Ashgari	2016	Hypertension Research	Non-clinical trials
Hirahatake	2016	Circulation	Dietary information not provided
Rebhold	2016	American Journal of Nephrology	Non-clinical trials
Mejia	2016	The FASEB Journal	Only abstract
Ashgari	2017	Nephrology, Dialysis, Transplantation	Non-clinical trials
Horikawa	2017	Nutrients	Non-clinical trials
Duncan	2017	Diabetology and Metabolic Syndrome	Dietary information not provided
